# Efficacy of Telephone Health Coaching Integration with Standard Multidisciplinary Care for Adults with Obesity Attending a Weight Management Service: A Pilot Study

**DOI:** 10.3390/nu13114078

**Published:** 2021-11-15

**Authors:** Sarah Driscoll, Gideon Meyerowitz-Katz, Golo Ahlenstiel, Tahlia Reynolds, Kate Reid, Ramy H. Bishay

**Affiliations:** 1 Metabolic and Weight Loss Program, Department of Endocrinology and Diabetes, Blacktown Hospital, Western Sydney Local Health District, Sydney, NSW 2148, Australia; Golo.Ahlenstiel@health.nsw.gov.au (G.A.); Ramy.Bishay@health.nsw.gov.au (R.H.B.); 2 Blacktown Clinical School, School of Medicine, Western Sydney University, Sydney, NSW 2148, Australia; 3 Western Sydney Diabetes, Integrated and Community Health Directorate, Blacktown Hospital Department of Endocrinology and Diabetes, Western Sydney Local Health District, Sydney, NSW 2148, Australia; Gideon.MeyerowitzKatz@health.nsw.gov.au; 4 Storr Liver Centre, Westmead Millennium Institute, Westmead Hospital, University of Sydney, Sydney, NSW 2148, Australia; 5 New South Wales Get Healthy Service, Centre for Population Health, Ministry of Health, Sydney, NSW 2148, Australia; Tahlia.Reynolds@health.nsw.gov.au; 6 Pregnancy and Adult Prevention Programs, Centre for Population Health, Ministry of Health, Sydney, NSW 2148, Australia; Kate.Reid2@health.nsw.gov.au

**Keywords:** complex obesity, multidisciplinary team, specialist obesity services, telehealth, weight loss

## Abstract

Australia has one of the highest prevalences of obesity in the developed world with recognised gaps in patient access to obesity services. This non-randomised before and after study investigated the health benefits and patient acceptability of integrating the Get Healthy Service, a state-funded telephone-delivered coaching service in Australia, as an adjunct to multidisciplinary care for adults attending a public obesity service. Forty-one participants received multidisciplinary care alone while 39 participants were subsequently allocated to receive adjunctive treatment with the Get Healthy Service. Weight, body mass index, glycosylated haemoglobin, measurement of hepatic steatosis and liver enzymes were collected at baseline and 6 months. Participant evaluation was obtained post intervention. Statistically significant reductions from baseline were achieved for both control and intervention with respect to weight (−6.7 ± 2.2 kg, *p* = 0.01; −12.6 ± 3.2, *p* = 0.002), body mass index (−2.3 ± 0.8, *p* = 0.01; −4.8 ± 1.2 kg/m^2^, *p* = 0.002) and glycosylated haemoglobin (−0.2 ± 0.2%, *p* = 0.2 (NS); −0.7 ± 0.2%, *p* = 0.02), respectively. There were no significant differences in steatosis or liver enzymes or in outcomes between control and intervention cohorts. A high level of patient acceptability was reported. Integrating telephone-delivered coaching provided non-inferior care and high levels of patient satisfaction. Telephone coaching aligned with the principles of an obesity service should be trialled to improve patient access to obesity interventions.

## 1. Introduction

Obesity (defined using a body mass index (BMI) ≥ 30 kg/m^2^) is a global epidemic with the worldwide prevalence increasing at an alarming rate [1]. Australia has one of the highest prevalences of obesity in the developed world with one in three Australian adults now classified as obese [2]. Obesity is associated with multisystem comorbidities such as Type 2 DM (T2D), metabolic associated fatty liver disease (MAFLD), cardiopulmonary diseases, musculoskeletal disorders, some cancers, decreased quality of life and early mortality [3,4].

While national guidelines recognise the key role that primary care plays in identifying and implementing lifestyle interventions to support overweight and obesity management [5,6], primary care-led interventions are unlikely to be adequate for patients with clinically severe obesity (BMI ≥ 40 kg/m^2^ or BMI ≥ 35 kg/m^2^ with comorbidities) and often complex health care needs [7,8,9].

Specialist obesity services (SOS), often found in public hospital systems, generally comprise of a multidisciplinary team (MDT) and are recommended for managing patients with severe obesity [3,5,6]. Specialist services have demonstrated potential to deliver clinically significant weight loss with associated improvements in health outcomes, obesity support as well as weight loss pharmacotherapy and bariatric surgery as appropriate [10]. Of the 16 public hospital obesity services available across Australia, patient access is limited by stringent entry criteria, prolonged wait lists and geographical location [11]. Additionally, many obesity services have inadequate staffing models to support the intensity of treatment and follow-up practices recommended for effective obesity management [5,7,11,12]. 

Telephone-delivered health coaching is a well-established care model to support dietary and lifestyle changes [13]. Not only does this modality offer the potential for a wide population reach, it also presents a cost-effective approach to provide the intensive, individualised contact necessary to promote behaviour change [14,15,16]. It is especially relevant during the recent global COVID-19 pandemic. Telephone-delivered coaching has shown benefits in supporting patients across a variety of domains such as increasing physical activity and promoting dietary behaviour changes [14,15,17,18,19,20], smoking cessation, promoting cancer screening and preventative behaviours for chronic diseases [21,22,23]. While telephone-delivered coaching has also shown promise in overweight and obesity management [24,25,26,27,28,29,30,31], the literature on its use as a tool for patients with severe obesity is limited [32]. 

The Get Healthy Information and Coaching Service (GHS) is a government-funded telephone-delivered information and coaching service available to adults within New South Wales (NSW), Australia. The GHS offers 10 telephone calls delivered over a 6-month period targeting healthy eating, physical activity and achieving and sustaining a healthy weight. Participants receive counselling from a personal health coach with university qualifications in dietetics, exercise psychology and/or psychology for the duration of the coaching period. Enhancement programs are also available for specific health conditions and populations and have been successfully integrated into routine care across several health settings. The Type 2 Diabetes Prevention Program has shown clinical improvements in anthropometric and lifestyle risk factors for adults at risk of T2D [33], The Get Healthy in Pregnancy (GHiP) Program has showed promise in supporting women across NSW achieve a healthy gestational weight and was well received by participants [34]. Finally, the Aboriginal Program was developed to effectively meet the health and cultural needs of Aboriginal communities and contributed to a reduction in the risk of chronic diseases for this population [35]. 

Despite the effectiveness of the GHS in supporting individuals’ efforts to achieve and sustain moderate positive behaviour changes and reductions in chronic disease risk factors [36], no research exists on its role in supporting patients with severe obesity as an adjunct to standard multidisciplinary care for adults attending a tertiary hospital SOS. As the frequency of contact with a health professional appears to positively influence the success of weight loss interventions in adults, best practice guidelines recommend weekly to fortnightly monitoring for the first 3 months with a plan for long-term monitoring for up to 2 years [5,7]. Due to resource limitations existing within the small number of SOS available within Australia, this intensity of treatment is not feasible [11]. It was envisaged that such an integration would reduce the finite resources for staff face-to-face clinical time in SOS, improve outcomes given the regular contact with patients and would have cost-savings and other quality benefits such as patient convenience.

This is the first study to investigate the health outcomes and patient acceptability of integrating telephone-delivered coaching using the GHS as an adjunct to MDT care for adults attending a SOS. Due to the association between obesity, T2D and MAFLD, secondary outcomes of this study included improvements in glycosylated haemoglobin (HbA1c), liver stiffness as assessed by FibroScan^®^, and liver enzymes alanine aminotransferase (ALT) and gamma-glutamine transpeptidase (GGT), which are commonly elevated in MAFLD. It is hypothesised that participants who complete the GHS coaching program as an adjunct to MDT care would achieve greater weight loss with associated greater reductions in HbA1c, liver stiffness, ALT and GGT enzymes when compared to participants who receive standard MDT care alone.

## 2. Materials and Methods

### 2.1. Participants and Study Design

Participants were recruited from the Blacktown Metabolic and Weight Loss Program, Blacktown-Mt Druitt Hospital, New South Wales, Australia. This SOS accepts patient referrals from any physician or specialist provided patients are over 18 years of age with Class III obesity (BMI ≥ 40 kg/m^2^) with two obesity-related complications (e.g., hypertension, hyperlipidaemia, obstructive sleep apnoea or other sleep-disordered breathing, non-alcoholic fatty liver disease, prediabetes, cardiovascular or cerebrovascular disease, joint disease, depression or anxiety, skin or soft tissue infections, urinary or faecal incontinence, venous thromboembolism, etc.) or Class II obesity (BMI ≥ 35 kg/m^2^) with co-existing T2D and no major mental health, drug or alcohol abuse/addiction. Study participants were consecutively recruited from May 2019 to October 2019. Participants who required individualised nutrition counselling were excluded from this study (e.g., participants with complex comorbidities unable to receive standard MDT care, cognitive impairment and language barriers).

**Figure 1:**
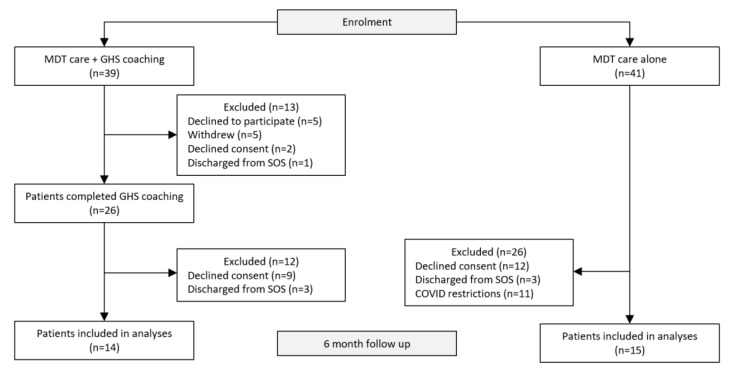
Study design and participation from recruitment to 6 months.

This non-randomised before and after study comprised two cohorts—from August 2019 to October 2019, 41 participants were subsequently recruited to receive MDT care alone and formed the control cohort (Figure 1). The intervention group comprised 39 participants and were recruited from May 2019 to July 2019 to receive GHS coaching over a 5-month period as an adjunct to MDT care. Recruitment occurred at different time points to reduce patient cross-over in patient support groups. Written informed consent was obtained from all study participants by research staff. This study was approved by the Western Sydney Local Health Research Ethics Committee.

### 2.2. Interventions

#### 2.2.1. Control

The control group consisted of one year of usual MDT care delivered by the SOS. The SOS is a physician-led program, providing a suite of specialist services delivered by an MDT of health professionals including a staff specialist endocrinologist, accredited practicing dietitians, a clinical nurse consultant and diabetic educator, certified psychologists and a physiotherapist.

Patients were referred by their primary care practitioners or medical specialists and triaged according to clinical risk. Patients attended program orientation delivered by the MDT followed by a medical work-up appointment with research staff and medical assessment with the specialist endocrinologist. Patients with complex diabetes were referred to a diabetes educator as required for the stabilisation of diabetes control (if glucose levels consistently above 10 mmol/L or who require the intensification of insulin therapy) prior to dietetic intervention.

Patients attended an initial dietitian-led group education session to commence a very low-calorie diet (VLCD, 800 kcal daily) consisting of a total meal replacement program or a low-calorie diet (LCD, 1000–1200 kcal daily) consisting of a partial meal replacement program for 6 months. The patients were provided the option between a VLCD or LCD to allow for individual goals and circumstances. The participants also had access to weekly dietitian-led patient support groups to allow for regular monitoring and counselling to improve adherence to dietary interventions, though data on compliance was not collected due to the expected bias from patient recall. Following this, study patients were transitioned into dietitian-led group programs focusing on a staged food reintroduction, dietetic counselling and weight maintenance support. Individuals in the study were also referred to psychologists for assessment and cognitive behavioural therapy group programs (e.g., patients with active or past history psychopathology); this comprised 6 1 h sessions in principles of emotional regulation, mindfulness, managing depression, anxiety and eating behaviours. Physiotherapy interventions were also included in the standard of care for high-risk patients (e.g., patients with chronic pain or mobility limitations, using walking aids) and referred for assessment and supervised exercise programs (8 × 1 h onsite gym session) and 6 educational 1 h group sessions. The interventions described occurred over the course of the patients one-year program enrolment, after which time the patient is discharged from the SOS back to primary care or a select number of patients are eligible to receive publicly funded bariatric surgery. The latter was decided based on the patient’s age, comorbidities, level of engagement and perceived benefit within a multidisciplinary case format discussion. Adherence to psychology and physiotherapy interventions was strongly advised, though data on adherence was not specifically collected.

#### 2.2.2. Intervention

The intervention group consisted of the MDT care described above with adjunct telephone-delivered coaching using the GHS. At the initial dietitian-led group education session, participants were provided with an overview of the GHS with the option to participate. Interested participants were referred to the GHS via a customised handover form. The standard GHS consists of 10 health coaching calls with enhancement programs offering up to 13 coaching calls delivered over a 6-month period. To provide the intensive contact required for this high-risk population, the enhanced call cycle protocol with a tapered schedule was utilised for this study. Participants were provided with the option to opt-out of the GHS at the screening call, after which participants who agreed to participate in the program were contacted by the GHS once a week for the first 6 weeks, with the following 7 phone calls completed on a fortnightly basis. The completion of the GHS coaching was defined as completing all 13 coaching calls—or if the participant reported they had reached their health goals, the option of early graduation was offered.

To ensure consistency of clinical practice guidelines, training was provided to the GHS coaches by the SOS dietitian prior to the intervention period. The GHS coaching therefore targeted the intensive dietary interventions set by the SOS in line with best practice for obesity management [5,6,7]. The content of the phone calls revolved around general counselling and support regarding adherence to the meal replacement program, increasing physical activity to meet national recommendations, optimising water intake, reduction in smoking and alcohol intake, healthy snacking and alternatives, improving mental health, adequate sleep and stress relief.

### 2.3. Outcome Measures

The primary outcome measure of this study was weight loss with weight and BMI changes obtained by dietitians using digital scales and a wall mounted stadiometer as part of patient support groups. To ensure consistency, patient support groups were scheduled at the same time each week and patients were advised to wear the same clothing and footwear. Secondary outcome measures included improvements in glycaemic control for those with T2D or prediabetes, liver stiffness, ALT and GGT enzymes and participant acceptability. For participants with prediabetes or T2D, HbA1c was measured by routine blood collections in addition to ALT and GGT enzymes and FibroScan^®^, which non-invasively measures liver stiffness as a marker of liver fibrosis and hepatic steatosis was performed by research staff to measure median stiffness and controlled attenuation parameter (CAP) median changes. Data collection for the outcomes described were collected at consent (baseline) and repeated at 6 months. Participant acceptability of the GHS was measured using semi-quantitative methods administered by a blinded evaluator at the end of the GHS intervention period. Participants scored their response on a Likert scale from 1 (strongly disagree) to 5 (strongly agree), in addition to open-ended questions included for free comment.

### 2.4. Statistical Analysis

Differences between parameters achieved in the control and intervention arms from baseline to 6 months in total body weight, BMI, HbA1c, FibroScan^®^ changes as well as ALT and GGT enzymes between control and intervention groups were calculated using a paired t-test. To determine differences in the same outcomes between the control and intervention arms, a mixed analysis of variance model (ANOVA) with Tukey’s tests were used to correct for multiple comparisons. A *p* < 0.05 was taken to be statistically significant. Data were computed using GraphPad Prism version 8.0.0 for Windows, GraphPad Software, San Diego, CA, USA). Responses to the questionnaires were calculated using mean +/− SD using Excel (Microsoft^®^ 2021).

## 3. Results

A total of 80 participants were eligible to participate in the study from 1 May 2019 to 31 October 2019 (Figure 1). Among these, the control arm consisted of 41 participants who received MDT care alone with 15 participants included in the final analyses. Thirty-nine participants were referred to the GHS coaching program as an adjunct to MDT care with 26 participants included in the final analyses. Participants received a total of 13 telephone coaching sessions lasting 8–33 min (average 14 min) over a 5-month period from the time of referral.

### 3.1. Participant Characteristics and Risk Factors

Table 1 and Table 2 present the characteristics and study parameters of participants at baseline and at 6 months. The sample comprised of adults primarily 40 years of age and above (78.6% intervention vs. 66.7% control), the majority being female (78.6% intervention vs. 66.7% control).

Compared to baseline, patients within the control group lost −6.7 ± 2.2 kg (150.8 ± 9.8 kg vs. 144.1 ± 10.2, *p* = 0.01) whereas those in the intervention arm lost −12.6 ± 3.2 kg (141.9 ± 9.9 kg vs. 129.3 ± 9.6, *p* = 0.002) at the end of the 6-month study period. The differences between both arms did not reach statistical significance (mean difference −5.9 ± 3.9 kg, *p* = 0.14; Table 2). The data were predictably similar for BMI in both control (−2.3 ± 0.8 kg/m^2^; 53.2 ± 2.9 kg/m^2^ vs. 50.9 ± 3.2, *p* = 0.01) and intervention (−4.8 ± 1.2 kg/m^2^; 52.1 ± 2.6 kg/m^2^ vs. 47.3 ± 2.5, *p* = 0.002), respectively, with no statistically significant changes between cohorts (mean difference −2.4 ± 1.4 kg/m^2^*, p* = 0.10).

With respect to glycaemic control, differences in HbA1c were seen post-intervention (6.5 ± 0.4% vs. 5.8 ± 0.2) with a mean absolute difference of −0.7 ± 0.2% (*p* = 0.02) in the intervention group. Similar (albeit non-significant) reductions were seen in the control group following the study intervention (−0.2 ± 0.2%; 6.7 ± 0.4 vs. 6.5 ± 0.5%, *p* = 0.24). Between-group differences did not reach statistical significance (mean difference −0.41 ± 0.32%, *p* = 0.22).

There were trends for reduction in FibroScan^®^ median stiffness for both control (9.1 ± 1.3 kPa vs. 6.8 ± 0.8, *p* = 0.22) and intervention (7.4 ± 0.8 vs. 5.9 ± 0.8, *p* = 0.07) at the end of the study period, but the differences between groups failed to reach statistical significance (*p* = 0.49). However, due to body habitus and large abdominal girths, FibroScan^®^ scores could only be reliably obtained for 21 participants across the sample. The same observation was seen for CAP (Table 2). ALT similarly decreased in both control (49.5 ± 7.6 vs. 48.3 ± 10.3 IU/L, *p* = 0.9) and intervention groups (39.0 ± 6.2 vs. 30.3 ± 2.2 IU/L, *p* = 0.2) although it did not reach statistical significance. GGT levels also decreased but failed to reach statistical significance in the both control (34.1 ± 4.5 vs. 34.5 ± 5.2, *p* = 0.9) and intervention groups (35.8 ± 4.7 vs. 31.7 ± 4.7, *p* = 0.3).

### 3.2. Participant Evaluation

Of the 14 patients who completed the GHS coaching, a 100% response rate was achieved and showed enthusiasm for the GHS coaching with strong participant satisfaction reported (Table 3). Participants reported that their GHS coach spoke to them in a manner they could understand and had the knowledge to provide guidance on the SOS nutrition interventions. This increased participant confidence and the ability to comply with the SOS nutrition interventions and enabled some participants to reduce the frequency of SOS clinic appointments. A clear link was found between the SOS and GHS which reassured participants that concerns could be escalated to the SOS when required. Participants attributed the success of the GHS to the personal connection that came from having the same GHS coach providing one-on-one coaching for the duration of the coaching period, which acted as an additional layer of support to SOS care.

## 4. Discussion

The integration of telephone-delivered coaching into an intensive-lifestyle MDT program did not yield significant differences in health risk factors when compared to intensive lifestyle MDT care alone. Clinical within-group improvements were demonstrated for weight loss, improvements in glycaemic control as well as reductions in liver stiffness. A high level of patient acceptability, however, was reported on the addition of telephone-delivered coaching to MDT care. This is an important finding and consistent with the literature which also found the support and rapport built by the GHS coaches increased participant adherence and motivation [37]. As discussed, service limitations across SOS within Australia prohibit the intensity of follow-up practices recommended in severe obesity [11], therefore the integration of telephone-delivered coaching may present an innovate and sustainable method of suitability supporting patients in conjunction with MDT care.

Despite being a public health priority, there is limited research on the role of telephone-delivered interventions for populations with severe obesity, with the majority of existing literature targeting populations in overweight (BMI 25–29.9 kg/m^2^) or class II and below obesity categories (BMI < 40 kg/m^2^) [32]. While comparisons can be drawn with a study by Lewis et al., who found similar positive results among participants attending a SOS with Class III obesity, the intervention consisted of telephone and text message support combined [38], while the present study investigated SOS care combined with telephone support alone. The findings of this research are considered novel, contributing to this under-represented population and cannot be directly compared to existing research.

While weight loss was observed across both groups, participants who completed the telephone-delivered coaching in addition to MDT care achieved 8.88% total weight loss while participants who received MDT care alone reported an average of just 4.44% total weight loss over the 20-week intervention period. This is an important finding as a weight loss of 5% or more of initial body weight is considered a successful and clinically meaningful weight reduction leading to a decreased risk for development or improvement of obesity-related risk factors for many patients [39]. While weight loss of less than 5% may lead to clinically meaningful reductions in some obesity-related risk factors, larger weight losses are likely to produce greater benefits [12]. Furthermore, as initial weight loss is a strong predictor for long-term weight loss, if patients are to sustain health benefits when challenged by weight regain, treatment strategies that are likely to produce weight loss that is greater than 5% are recommended [40]. Due to the limited access of publicly funded bariatric surgery [11], many SOS mandate a 10% preoperative weight loss due to associations with increased postoperative weight loss, reduced operative time, surgical complications and mortality as well as serving as a method for patient selection [41,42,43]. While there was no significant difference found between groups, the addition of a telephone-delivered coaching program to MDT care may support patients’ efforts to meet this increasingly mandated preoperative weight loss requirement despite resource limitations.

Previously considered a permanent and progressive disease requiring lifelong treatment, the *DiRECT* trial demonstrated that a structured and intensive weight management intervention can lead to sufficient weight loss and T2DM remission, with results maintained over 2 years [44,45]. The dietary interventions from the *DiRECT* study are comparable to the present study, consisting of a flexible VLCD, stepped food reintroduction and structured support for long-term weight loss maintenance. While HbA1c reductions were observed across both groups in the present study, participants who completed the telephone-delivered coaching in addition to MDT care achieved significant reductions in HbA1c as opposed to participants who received MDT care alone, strengthening the beneficial role of the GHS in populations at risk of and with established T2D.

In parallel to the obesity epidemic, MAFLD has become a global health hazard, leading to inflammation and fibrosis, cirrhosis, liver failure, hepatocellular carcinoma and early mortality [46]. Lifestyle interventions that promote weight loss and physical activity are recommended as first-line treatment [47]. The higher weight loss achieved in participants who completed the telephone-delivered coaching similarly experienced greater reductions seen in in ALT and GGT compared to those who received SOS care alone, although these differences were non-significant. Notably, the patients were only monitored for six months and changes on FibroScan^®^ may require longer to be fully appreciated. These results substantiate prior studies on the effect of telephone-delivered interventions on increased self-efficacy in adherence to diet, physical activity, and healthy behaviours [48], as well as obesity-related liver enzymes in patients with MAFLD [47].

There are several limitations to this study. Firstly, this was a non-randomised before and after study with the recruitment of the two cohorts occurring at different time points. Seasonal differences, holidays and the global COVID-19 pandemic may have impacted compliance to interventions and access to MDT care. Participant compliance with intensive dietary interventions also relied upon self-reported data obtained in weekly patient support groups. In addition, while participants who completed telephone-delivered coaching achieved higher reductions across all primary outcomes, the differences were not significant due to the small sample size. Only participants who graduated from GHS coaching and remained part of the SOS at 6 months were included in the follow-up data collection and analysis. As the sample sizes were small, this limits the generalizability of the findings and made it impossible to perform intention-to-treat analyses. This may reflect a biased, highly motivated group. While the study duration was appropriate to investigate early weight loss, long-term follow-up is required to investigate the effect of adjunct telephone-delivered coaching on long-term obesity management; however, the trends across the primary outcomes in this small pilot study provide reassurance of the utility of this program. More clinically significant results may be obtained with a trial of a longer duration. Beyond gender and age, no further patient demographics were collected as part of this study with recommendations for future research to investigate the effect of demographics such as ethnicity, education levels and socioeconomic status on outcome measures. Furthermore, controlling for baseline body weight, the time of year of intervention, ethnicity and physical activity was not possible due to COVID-19 restrictions at the time of the study.

## 5. Conclusions

No significant differences in health risk factors were found with the integration of telephone-delivered coaching as an adjunct to MDT care for adults attending a public SOS. The high level of patient acceptability, however, indicates that telephone coaching aligned with the principles of an obesity service should be trialled to improve patient access to obesity interventions and is especially relevant in the Australian setting with many patients living in remote or in rural areas, especially in the current time of a global pandemic, which imposes challenges on many health services.

## Figures and Tables

**Table 1:** Participant characteristics at baseline.

Characteristics	MDT Care Alone		MDT Care + GHS Coaching	
	*n*	%	*n*	%
Gender				
Female	10	66.7	11	78.6
Male	5	33.3	3	21.4
Age				
18–39	5	33.3	3	21.4
≥40	10	66.7	11	78.6

**Table 2:** Participant risk factors at baseline and 6 months post-intervention.

Parameter	MDT Alone		MDT + GHS Coaching		*p*
	Baseline	6 Months	Baseline	6 Months	
Weight (kg), *n*	*n* = 15		*n* = 14		
	150.8 ± 9.8	141.9 ± 9.9	141.9 ± 9.9	129.3 ± 9.6 ^#^	0.14
BMI (kg/m^2^), *n*	*n* = 15		*n* = 14		
	53.2 ± 2.9	53.2 ± 2.9	52.1 ± 2.6	47.3 ± 2.5 ^#^	0.10
HbA1c, *n*	*n* = 15		*n* = 14		
Mean (%) mmol/mol	6.7 ± 0.4 50.2 ± 4.3	6.7 ± 0.4 50.2 ± 4.3	6.5 ± 0.4 47.0 ± 4.0	5.8 ± 0.2 ^#^ 39.7 ± 2.2 ^#^	0.22
FibroScan^®^ Median stiffness, *n*	*n* = 11	*n* = 11	*n* = 13	*n* = 10	
	9.1 ± 1.3	9.1 ± 1.3	7.4 ± 0.8	5.9 ± 0.8	0.49
FibroScan^®^ CAP median, *n*	*n* = 11	*n* = 11	*n* = 13	*n* = 10	
	349.9 ± 11.9	349.9 ± 11.9	347.9 ± 15.1	320.8 ± 23.0	0.30
ALT, *n*	*n* = 15		*n* = 14		
	49.5 ± 7.6	49.5 ± 7.6	39.0 ± 6.2	30.3 ± 2.2	0.47
GGT, *n*	*n* = 15		*n* = 14		
	34.1 ± 4.5	34.1 ± 4.5	35.8 ± 4.7	31.7 ± 4.7	0.41

Abbreviations: BMI, body mass index; HbA1c, glycosylated haemoglobin; ALT, alanine aminotransferase; *GGT*, gamma-glutamine transpeptidase. *p* < 0.5 was considered statistically significant. ^#^ *p* < 0.05 vs. baseline for within group differences. Right column shows *p* values for between group differences. All values shown as mean +/− standard error of the mean.

**Table 3:** Qualitative data from participant evaluation.

Theme	Question	M	SD	Quotation
Coach communication	My GHS coach spoke to me in a way that I could understand	4.8	0.43	“The GHS is fantastic. My coach knew the SOS so well and supported everything I did”.
Participant confidence	The support from the GHS increased my confidence to manage my health goals	4.4	0.84	“My GHS coach was great. She helped me to keep on track of my appointments and gave me the confidence in myself to continue on my nutrition programs. My coach also called on time, so I knew when to expect the phone calls which was helpful for me to set the time aside at work”. “My coach was very patient and provided me with confidence and reassurance if I got off track”.
Participant ability	The support from the GHS increased my ability to follow my nutrition programs	4.4	0.76	“Great service really helped to keep me accountable between my SOS clinic appointments”. “I was very happy with my GHS. I found it so motivating between my clinic appointments, it was all I needed to keep on track and continue with my program”.
Appointment frequency	The support from the GHS allowed me to reduce the frequency of my clinic appointments	3.3	1.33	“The fact that the phone calls were a scheduled appointment really kept me on track. I would reserve the time for the call, save up my questions and ask my coach. It allowed me to rely less on the SOS which was handy as my mobility isn’t the best”. “The GHS enabled me to stay on track in between my appointments at the SOS and also helped me to cut down coming into the clinic so frequently to ask small questions about my program. I had such a positive experience, great idea to include this as part of the service”. “Working full time, I found the phone coaching really valuable. It allowed me to cut down my face-to-face appointments at the SOS during my coaching period”.
Coach understanding	My GHS coach understood the SOS nutrition programs	4.1	1.17	“My GHS coach was always willing to go that extra mile. I once had a question about allowable vegetables as part of my program while my coach didn’t know at that time, within 5 min he had called me back with the answer. Great service”.
Coach knowledge	My GHS coach provided knowledge and guidance on my nutrition programs	4.5	0.76	“Although I do need the accountability of getting on scales to be weighed and asking questions face to face, just having someone check in on your progress, give you tips and ideas to keep on track made all the difference”.
Ability to escalate	If I had a problem with my nutrition program my GHS coach could provide me with advice or escalate my concerns to the SOS	4.4	0.94	“The GHS was great. I felt there was a clear link between the services, and everyone was working together. There was a time where I had a specific question relating to my health and program, the GHS directed me back to the SOS and informed the SOS who then followed up my concern. Great service”.
Link between services	I felt there was a clear link between the GHS and SOS	4.4	0.85	“The whole process worked really well. Everyone was on the same page it felt like they all worked together. It was great to have the option of evening calls as I work full time. Keep this up”. “The GHS staff were invaluable. There was such a great link between the SOS and GHS, if I had a question about my program the GHS staff could literally direct me to the exact page of the SOS resource I needed! It was great”! “The GHS was great. I felt there was a clear link between the services, and everyone was working together. There was a time where I had a specific question relating to my health and program, the GHS directed me back to the SOS and informed the SOS who then follow up my concern. Great service”.

Abbreviations: M, mean; SD, standard deviation. Rating scale: 5 = strongly agree, 4 = agree, 3 = neutral, 2 = disagree, 1 = strongly disagree.

## Data Availability

Not applicable.
